# The Effects of Nurse-Led Multidisciplinary Team Management on Glycosylated Hemoglobin, Quality of Life, Hospitalization, and Help-Seeking Behavior of People with Diabetes Mellitus

**DOI:** 10.1155/2019/9325146

**Published:** 2019-12-21

**Authors:** Yunxia Ni, Suzhen Liu, Jiping Li, Ting Dong, Lin Tao, Li Yuan, Meilan Yang

**Affiliations:** ^1^West China Hospital/West China School of Nursing, Sichuan University, Chengdu, Sichuan Province, China; ^2^Department of Endocrinology, West China Hospital, Sichuan University, Chengdu, Sichuan Province, China; ^3^Yulin Community Health Service Center, Chengdu, Sichuan Province, China

## Abstract

**Aim:**

To evaluate the effect of community-nurse-led multidisciplinary team management on glycosylated hemoglobin (HbA1c), quality of life (QOL), hospitalization, and help-seeking behavior in people with type 2 diabetes mellitus (DM).

**Methods:**

A quasi-experimental trial was conducted among people with type 2 DM from two community centers in China. The intervention group (*n* = 88) received community-nurse-led multidisciplinary team management for 2 years, while the control group (*n* = 91) received usual care. Data regarding HbA1c, QOL (assessed by the SF-36), hospitalization, and help-seeking behavior were collected at baseline and at 6, 12, and 24 months.

**Results:**

During the 24-month project, the intervention group demonstrated 1.08% reduction in HbA1c, whereas the control group achieved an increase of 0.45%. The differences between the two groups were statistically significant (*P* < 0.001). The intervention group showed greater increased in QOL scores (from 66.43 to 70.47, *P* < 0.001), more decrease in hospitalization (OR = 2.981, 95% CI: 1.016, 8.752 versus OR = 1.189, 95% CI: 0.411, 3.444; *P* = 0.028) when compared with the control group. The percentage increase of seeking help from nurses in the intervention group (from 12.5% to 57.3%, *P* < 0.001) was significantly greater than that in the control group after the intervention.

**Conclusions:**

Nurse-led multidisciplinary team management is an effective intervention for improving glycemic control, QOL, hospitalization, and help-seeking behavior for people with DM in a community.

## 1. Introduction

Diabetes mellitus (DM) is one of the most common chronic diseases in the world. There are more than 463 million diabetics worldwide at present, and with the incidence of this disease increasing, this number is expected to exceed 700 million by 2045 [1]. China has the largest number of diabetics, and the prevalence of DM has been conservatively reported to be approximately 11.6% [2]. Due to the insidious progress of microvascular and macrovascular complications in DM, patients may go undetected, resulting in multiple complications, including retinopathy, nephropathy, neuropathy, limb amputation, and stroke [3–5], which can lead to a serious social and economic burden [6, 7].

Previous research has suggested that community-based adaptions of DM intervention programs can help patients achieve meaningful glycemic decreases and healthy lifestyles at a lower overall cost [8, 9]. Currently, most type 2 diabetics receive their care from primary care physicians, resulting in an increasingly heavy workload for physicians [10]. Primary care physicians have typically performed various duties, thus leading to time-limited visits for patients [11]. In addition, the nurses' autonomy and level of intervention depended on the primary care physician. Hence, numerous interventions have been explored to address the challenges of inadequate control with increasing patients [12–14]. As a consequence, many researchers extended the role of nurses by instituting them as care managers [15–17].

Studies of nurse-led management for people with DM have shown significant improvement in self-management and clinical outcomes [15, 16, 18]. Sadly, according to some studies, the leadership role of nurses has been overemphasized, leading to neglect of the importance of multidisciplinary teams. Quite a few teams were only made up of nurses and community health workers, sometimes including primary care physicians or pharmacists rather than multidisciplinary teams [15, 16, 19]. Even for multidisciplinary team management, the central role was usually played by primary care physicians or specialists instead of nurses [20–22]. Furthermore, the duration of follow-up was usually less than one year, long-term effects of nurse-led multidisciplinary team were scarce [19, 23]. Although patients were uniquely qualified to say whether the intervention was acceptable or suitable for them [24, 25], most studies only focused on the effects of clinical outcomes or self-care. Thus, evaluating the effects from the distinctive perspective of patients was essential to better understand the patients' acceptance. Hence, our study explored the effects of help-seeking behavior changes after the nurse-led management.

This study aimed to evaluate the effect of nurse-led multidisciplinary team management on hospitalization reduction, help-seeking behavior changes, and quality of life (QOL) in patients with type 2 DM for 24 months.

## 2. Methods

### 2.1. Design

People with type 2 DM were recruited by convenience sampling from outpatient department of Yulin and Tiaosanta Community Centers in Wuhou District, Chengdu City, China. For this quasi-experimental study, participants were assigned based on their residence address: patients who came from Yulin Community Center were assigned to the intervention group and those who came from Tiaosanta Community Center were assigned to the control group. This prospective clinical trial was conducted from 2014 to 2016.

### 2.2. Participants

The inclusion criteria of participants were as follows: (1) documented diagnosis of type 2 DM; (2) age ≥ 35 years old (governmental requirement for management in community centers); (3) voluntary participation in the study; (4) ability to communicate; and (5) availability for contact by telephone at home. The exclusion criteria were as follows: (1) current pregnancy or planned pregnancy; (2) serious complications or comorbidity; (3) limitation of activities; (4) mental disorders; (5) recent cardiovascular event (<6 months before inclusion); and (6) simultaneous participation in other research studies.

Our sample size was based on the following assumptions. Assuming a 2% glycosylated hemoglobin (HbA1c) decrease in the intervention group, a 0.8% HbA1c decrease in the control group, and a power level of 95%, a sample of 71 patients was estimated. We estimated a 20% discontinuation rate, and ultimately, a required sample size of 86 was calculated for each group.

### 2.3. Intervention

Nurse-led multidisciplinary team management consisted of three main intervention modes:

1. A series of health education classes delivered in a group education format
2. Individualized counseling via telephone and face-to-face follow-up visit
3. Pamphlet and self-monitoring workbook were hand out

The intervention group received nurse-led multidisciplinary team management for 2 years. The multidisciplinary team was composed of large teaching hospital specialists (endocrinologists, dietitians, psychologists, cardiologists, nephrologists, and specialist nurses), one general practitioner, and four community nurses. Nurses went through a training program that included theoretical input (20 hours), practical training with endocrinology in the hospital (1 month), individual self-study, and review (varying lengths of training). The community nurse not only played a central role on the team in developing and conducting a patient-specific management plan but also served as a liaison between participants and primary care physicians. Nurses and primary care physicians provided referrals to a specialist service for participants.

Periodic group education involved about monthly 90-minute sessions organized by nurses (6 months as one period over the course of 4 periods, five sessions per period). Thus, a total of 20 group education sessions were performed during 2 years. Session topics included a definition of diabetes, target ranges for essential results (glucose, blood pressure, lipids, weight), basic nutrition concepts, exercise strategies, medicine adherence, self-monitoring of blood glucose, smoking cessation, symptoms of acute and chronic complications, and emotion management. The contents of the classes were similar over all four periods but became more in-depth and practical over time to reinforce self-management skills.

Nurses tracked participants' self-management progress to determine the delivered dose of the intervention. Participants who reached the goal made by nurses and patients themselves received one telephone follow-up visit and one face-to-face individual counseling each month, to identify barriers and issues, assist in problem-solving, and provide feedback to primary care physicians. Those participants not making progress toward their goal levels received more frequent telephone follow-ups or home visits on average twice a month. Documents designed to record follow-up information for every participant were important to ensure intervention fidelity and provided a place to take notes on participants' goals, potential barriers, management plans, and strategies to overcome difficulties. This work was completed during the counseling or follow-up sessions. For participants suffering from serious problems (for instance, psychological problems, retinopathy, and nephropathy), nurses referred them to other members of the multidisciplinary team and kept track of the problem.

Participants also received a pamphlet developed specifically for the program as a guide to self-care (strategies for caloric control, physical activity, customized tips for taking medicine, and mental accommodation). They also received a self-monitoring workbook to record their daily nutrition intake, activities, and blood glucose.

Participants in the control group received usual care from their primary care physician, including educational classes 2 to 3 times per year, on average, and routine face-to-face follow-up at least 4 times per year. Medications were prescribed and adjusted based on the sole clinical judgment of the physician. Aside from performing the BP and blood glucose measurements when patients came to the community center for office visits, nurses rarely participated in DM management.

### 2.4. Data Collection

Data collection consisted of three parts as follows: (1) baseline demographic information collected through interviewing the patient was completed before the intervention; (2) the QOL, hospitalization, and help-seeking behavior were completed before, 6 months, 12 months, and 24 months after the intervention; and (3) HbA1c levels were measured before, 6 months, 12 months, and 24 months after the intervention.

#### 2.4.1. HbA1c

HbA1c assays were conducted in the same laboratory using latex agglutination turbidimetric method with Hitachi 7600-020 automatic biochemical analyzer (Hitachi Company, Tokyo).

#### 2.4.2. Questionnaires

Questionnaires used in this study consisted of researcher-designed questionnaire regarding demographic, hospitalization, help-seeking behavior, and the 36-item Short-Form Health Survey (SF-36) for the QOL. The SF-36 questionnaire provides a profile of eight subscales that measure physical functioning, role-physical, bodily pain, general health, vitality, social functioning, role-emotional, and mental health. A 0–100 standardized score is ultimately presented. The higher score indicates better health conditions. The validity and reliability of this questionnaire were evaluated in Chinese population and reported the Cronbach's *α* coefficient ranging from 0.77 to 0.88 [26]. Hospitalization data were obtained through patient-reported number of hospitalizations caused by diabetes during the past 6 months. Help-seeking behaviors were identified via one question “when you have problems related to diabetes, who are you seeking for help?” Patients made choices from the following options: doctors, nurses, or others. Multiple choices were accepted.

### 2.5. Statistical Analysis

Data were analyzed by the SPSS version 19.0 software (IBM Corporation, Armonk, NY, USA). Statistical tests were used to study differences in baseline demographic with a *t* test used for continuous variables and a chi-squared test for categorical variables. The primary outcomes were changes from baseline to 24 months in HbA1c, QOL, hospitalizations, and help-seeking behaviors. General linear mixed models (for continuous variables) and generalized estimating equations (GEE) (for categorical variables) were used to examine the effectiveness of the program, controlling for the covariates of age, gender, diabetes complications, and treatment modality. Intention-to-treat (ITT) analyses were conducted, and values were imputed to replace missing data, as indicated previously. A significance level of 5% (two-tailed) was used for all tests.

### 2.6. Ethical Considerations

This study was approved by the ethics committee of West China Hospital of Sichuan University (no. 2015-110). Information and explanation of the ethical observations of the study were provided to the subjects, and they were asked to sign a consent form. The patients were free to withdraw from the study at any time, and all data were maintained confidentially.

## 3. Results

### 3.1. Participant Demographic Characteristics

**Figure 1:**
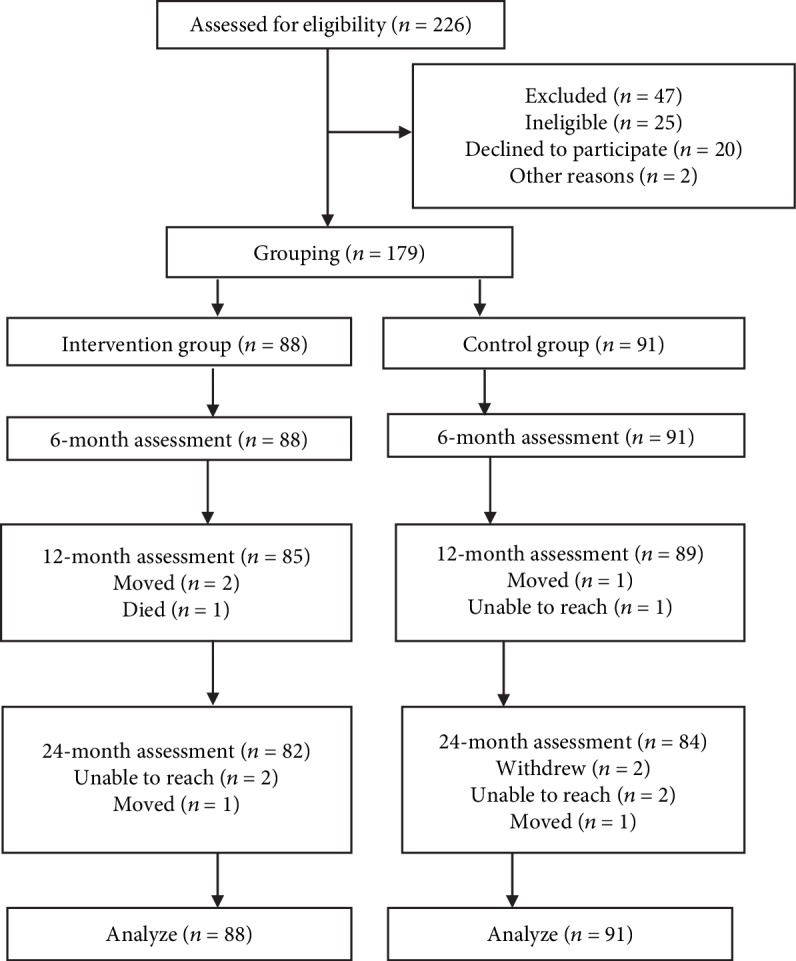
Flow diagram of participants through each stage of the trial.

One hundred seventy-nine participants were enrolled in this study: 88 in the intervention group and 91 in the control group.  Figure 1 shows the flow of patients through the study. In total, 13 patients (7.3%) were lost to follow-up (6 patients came from the intervention group and 7 from the control group). The characteristics of the participants are shown in Table 1. The intervention and control groups were well matched on most variables with the exception of age distribution, HbA1c, QOL, and hospitalizations. Nearly half patients (46.6%) in the intervention group were older than 70 years; however, 49.4% of patients in the control group were between 60 and 70 years old. Age distribution could be operating in unmatched HbA1c, QOL, and hospitalizations.

### 3.2. HbA1c and QOL

The HbA1c in the intervention group decreased from 7.08% to 6.03%, with a reduction of 1.08%, while the control group showed an increase of 0.45% (Table 2). The differences between the two groups were statistically significant (*P* < 0.001). Mean score changes of QOL in the intervention group showed greater increased than those in the control group (*P* < 0.001) (Table 2).

### 3.3. Hospitalizations

The intervention group had nearly triple the risk of the hospitalizations compared with those in the control group before the intervention (Table 3). The odds ratio of hospitalizations decreased to 1.189 after the intervention. There was a statistically significant difference regarding the hospitalizations changes between two groups according to the generalized estimating equations (Wald *χ*^2^ = 4.83, *P* = 0.028).

### 3.4. Help-Seeking Behavior

When patients have difficulty in controlling diabetes, most of them seek help from doctors firstly instead of nurses for both groups at baseline (Table 4). At 24 months, 57.3% patients reported that they were willing to seek help from nurses in the intervention group. This percentage increase of seeking help from nurses in the intervention group was significantly greater than the increase in the control group (44.8% versus 8.1%, *P* < 0.001). The percentages of seeking help from doctors were decreased for both groups, but there was no statistically significant.

## 4. Discussion

The results of our study revealed that the nurse-led multidisciplinary team management leads to significant improving effects on HbA1c, QOL, hospitalizations, and help-seeking behavior changes. The study has several design and community-based methodological strengths. The intervention lasted for 2 years, allowing us to document the sustained effects. In addition, we first tested the intervention effects related to the participants' help-seeking behavior in their truest sense. Moreover, the community was close with local large teaching hospital and local health-care providers, resulting in timely care delivered to the participants and effective communication with team members. Furthermore, nurses as the core of the team were they themselves are part of the community, making it feasible to reform routine management in the future.

Although the control group showed a good glucose control and remained excellent until the end of the study, the intervention group reduction rates were especially noteworthy. The reductions in the intervention group were sustained over the course of 24 months, with evidence of further potential reductions if the intervention continued beyond the study period. Furthermore, the HbA1c reductions in the intervention group were not only statistically significant but also clinically significant. At 6 months after the intervention, the HbA1c in the intervention group has reached Chinese guideline goals (<7.0%), followed by sustained improvement during the 18-month maintenance period, which strongly suggests a clinical impact of the nurse-led team management. The results of our study were consistent with the results of other studies showing that nurse-led management has an improving impact on the HbA1c of the patients with type 2 DM [27–29]. However, it is noticeable that these studies had been only performed by nurses or nurse-community health workers team, while the management team of the current study was made up by multidisciplinary specialists. In a meta-analysis of 34 trials by nurse-led self-management education, the HbA1c level decreased by a mean of 0.7% [30], which showed less decline than our study. This difference could be explained by the duration of follow-up of the studies involved in the meta-analysis being usually less than 1 year, while our study performed the longer term to see the intervention impact.

Our findings confirmed the findings of previous studies [28, 31, 32] that nurse-led management improved the QOL of people with DM. However, the improvement had not been found in a trial by Blackberry et al. [16], which was in contrast to our findings. A possible reason for negative result may be the low intensity of their coaching and low patient participation. Ideally, nurses were supposed to provide eight telephone follow-up visits and one face-to-face coaching for each patient. In fact, patients only received a median of three telephone calls; what is more, a quarter of patients in the intervention group did not receive any telephone coaching. In our study, a more intensive intervention with more frequent calls and face-to-face counselling and with longer interaction was performed, helping to highlight the effect of the intervention.

Despite the hospitalizations of participants in the intervention group, they showed a higher risk than those in the control group; the risk of hospitalizations had significant reduction, which demonstrated that the nurse-led management was effective on hospitalization reduction, keeping with the findings from previous studies [33–35]. Davidson et al. assessed the hospitalizations before and after the intervention, without control group; moreover, the program was performed in a minority population [33], which was different from our study. We set up a control group and evaluate the intervention effect using the generalized estimation equation, showing rigorous methodology, further revised, and replenished previous studies.

The help-seeking behavior has changed after the intervention, and more percentage of the participants in the intervention group compared with the control group were willing to seek help from nurses. To some extent, it suggested that nurses get more trust from participants. Only when participants trust in the nurses' experience with DM management will they seek help from nurses [36, 37]. On the other hand, although the percentage reduction of seeking help from doctors showed no statistical significance, the overall trend of reduction was sustained. This trend revealed that some DM-related problems patients suffered could be solved by nurses [38], and there was no need to seek help from doctors, contributing to utilizing health-care resources efficiently, evolving the role of nurses appropriately [39, 40], and relieving the pressure of health care finally [41, 42]. Moreover, the help-seeking behavior changes demonstrated the acceptance of nurse-led management. In a cross-section study Lutfiyya et al., they analyzed the Medicare claims data, people with DM in the nurse practitioner only group had significantly improved outcomes compared with all primary care physician provider groups regarding health-care services utilization [43]. Consequently, nurse-led management for DM in primary setting was associated with more proper health care utilization.

This study has several limitations. First, to avoid contamination of intervention effects between groups, we allocated patients to groups by geographical distance of two community centers, with nonrandomized method. Second, this study was a single-center program in only one region, and further research can be conducted in multiple centers. Finally, the hospitalizations were patient-reported, which may have led to recall bias. Future studies should consider addressing this bias through medical records to collect data.

## 5. Conclusions

The implementation of nurse-led team management proves to be practical and feasible in community settings and is accompanied by favorable HbA1c, improved QOL, hospitalizations, and help-seeking behavior. It expanded the role of nurses in helping DM achieving an excellent glucose control, seeking help reasonably. In addition, we consider the intervention as a new approach for Chinese community management to address the shortage of primary care physicians. It is not known whether the nurse-led management has impact on cost-effectiveness in patients with DM.

## Figures and Tables

**Table 1:** Patient demographic characteristics at baseline.

Variable	All	Intervention	Control	*P* value
	(*N* = 179)	(*n* = 88)	(*n* = 91)	
Age, y, mean (SD)	66.5 (8.9)	66.4 (10.6)	66.6 (7.0)	0.866
Age, y, *n* (%)				0.014
<50	7 (3.9)	6 (6.8)	1 (1.1)	
50~<60	28 (15.6)	16 (18.2)	12 (13.2)	
60~<70	70 (39.1)	25 (28.4)	45 (49.4)	
≥70	74 (41.3)	41 (46.6)	33 (36.3)	
Gender, *n* (%)				0.610
Female	95 (53.1)	45 (51.1)	50 (54.9)	
Male	84 (46.9)	43 (48.9)	41 (45.1)	
Spouse, *n* (%)				0.948
Yes	165 (92.2)	81 (92.0)	84 (92.3)	
No	14 (7.8)	7 (8.0)	7 (7.7)	
Education level, *n* (%)				0.397
No schooling	35 (19.5)	16 (18.1)	19 (20.8)	
Junior/middle school	51 (28.5)	21 (23.9)	30 (33.0)	
High school	56 (31.3)	32 (36.4)	24 (26.4)	
Some college or above	37 (20.7)	19 (21.6)	18 (19.8)	
Employment status, *n* (%)				0.133
Employed	20 (11.2)	13 (14.8)	7 (7.7)	
Retired or unemployed	159 (88.8)	75 (85.2)	84 (92.3)	
Basic medical insurance, *n* (%)				0.240
Yes	177 (98.9)	86 (97.7)	91 (100.0)	
No	2 (1.1)	2 (2.3)	0 (0.0)	
Additional medical insurance, *n* (%)				0.754
Yes	122 (68.2)	59 (67.0)	63 (69.2)	
No	57 (31.8)	29 (33.0)	28 (30.8)	
Monthly income, *n* (%)				0.308
¥≤1500	30 (16.7)	14 (15.9)	16 (17.6)	
¥1501-4500	146 (81.6)	74 (84.1)	72 (79.1)	
¥>4500	3 (1.7)	0 (0.0)	3 (3.3)	
Diabetes complications				
Yes	70 (39.1)	39 (44.3)	31 (34.1)	0.160
No	109 (60.9)	49 (55.7)	60 (65.9)	
Treatment modality				
Diet/exercise	6 (3.4)	2 (2.3)	4 (4.4)	0.286
Oral medication	137 (76.5)	64 (72.7)	73 (80.2)	
Insulin therapy	8 (4.5)	6 (6.8)	2 (2.2)	
Oral medication and insulin therapy	28 (15.6)	16 (18.2)	12 (13.2)	
HbA1c, %, mean (SD)		7.08 (1.26)	6.34 (1.02)	<0.001
QOL, mean (SD)		66.43 (14.07)	74.71 (14.09)	<0.001
Hospitalizations, *n* (%)		13 (14.8)	5 (5.5)	0.039

SD: standard deviation; HbA1c: glycosylated hemoglobin; QOL: quality of life.

**Table 2:** Changes in HbA1c and QOL scores between the two groups.

Outcomes	Intervention group (mean ± SD)	Control group (mean ± SD)	Estimated between-group difference^∗^ (95% CI)	*P* value^Δ^
Change in HbA1c (%)	-1.08	0.45	-1.53 (-1.91, -1.14)	<0.001^⋄^
Baseline	7.08 ± 1.26	6.34 ± 1.02	0.74 (0.41, 1.09)	
6 months	6.72 ± 0.97	6.14 ± 1.02	0.58 (0.28, 0.87)	
12 months	6.22 ± 1.46	6.26 ± 1.17	-0.04 (-0.44, 0.36)	
24 months	6.03 ± 1.02	6.68 ± 1.48	-0.65 (-1.04, -0.26)	
Change in QOL score	4.04	-5.10	9.14 (1.21, 17.07)	<0.001^⋄^
Baseline	66.43 ± 14.07	74.71 ± 14.09	-8.27 (-12.43,-4.12)	
6 months	71.57 ± 14.65	74.89 ± 12.62	-3.31 (-7.34, 0.71)	
12 months	72.82 ± 13.90	73.45 ± 13.26	-1.18 (-6.56, 4.21)	
24 months	70.47 ± 13.75	69.61 ± 14.43	0.86 (-6.24, 7.97)	

SD: standard deviation; HbA1c: glycosylated hemoglobin; QOL: quality of life; CI: confidence interval. ^∗^Reference is the control group; ^Δ^intention-to-treat analysis using general linear mixed model with group; time, group × time effects, and covariates of age, gender, diabetes complications, and treatment modality. ^⋄^Overall effect *P* value.

**Table 3:** Changes in hospitalizations between the two groups.

Hospitalizations, *n* (%)	Intervention group	Control group	OR	95% CI	GEE	
					Wald *χ*^2^	*P* value^∗^
Baseline	13 (14.8)	5 (5.5)	2.981	1.016-8.752	4.83	0.028^Δ^
6 months	12 (13.6)	7 (7.7)	1.895	0.709-5.061		
12 months	10 (11.8)	4 (4.5)	2.833	0.853-9.411		
24 months	8 (9.8)	7 (8.3)	1.189	0.411-3.444		

OR: odds ratio; CI: confidence interval; GEE: generalized estimating equations. ^∗^Generalized estimating equations were used to analyze, with covariates of age, gender, diabetes complications, and treatment modality. ^Δ^Intervention effect *P* value.

**Table 4:** Changes in help-seeking behavior between the two groups.

Help-seeking behavior, *n* (%)	Intervention group	Control group	GEE	
			Wald *χ*^2^	*P* value^∗^
Percentage change in help-seeking from doctors	-10.4	-1.1	1.25	0.263^Δ^
Baseline^#^	65 (73.9)	66 (72.5)		
6 months	66 (75.0)	69 (75.8)		
12 months	59 (69.4)	65 (73.0)		
24 months	52 (63.4)	60 (71.4)		
Percentage change in help-seeking from nurses	44.8	8.1	19.36	<0.001^Δ^
Baseline^#^	11 (12.5)	11 (12.1)		
6 months	28 (31.8)	16 (17.6)		
12 months	35 (41.2)	17 (19.1)		
24 months	47 (57.3)	17 (20.2)		
Percentage change in help-seeking from others	0.1	-6.6	0.05	0.832^Δ^
Baseline^#^	15 (17.0)	19 (20.9)		
6 months	19 (21.6)	20 (22.0)		
12 months	23 (27.1)	18 (20.2)		
24 months	14 (17.1)	12 (14.3)		

GEE: generalized estimating equations. ^∗^Generalized estimating equations were used to analyze, with covariates of age, gender, diabetes complications, and treatment modality. ^Δ^Intervention effect *P* value; ^#^there were no significant differences between the two groups at baseline.

## Data Availability

The data used to support the findings of this study are available from the corresponding author upon request.
